# Smad transcription factors as mediators of 7 transmembrane G protein-coupled receptor signalling

**DOI:** 10.1038/s41401-024-01413-6

**Published:** 2024-11-06

**Authors:** Zheng-Jie Chia, Hirushi Kumarapperuma, Ruizhi Zhang, Peter J. Little, Danielle Kamato

**Affiliations:** 1 Institute for Biomedicine and Glycomics, Griffith University, Nathan, QLD Australia; 2 School of Pharmacy, The University of Queensland, Woolloongabba, QLD Australia; 3 School of Environment and Science, Griffith Sciences, Griffith University, Nathan, QLD Australia; 4 Department of Pharmacy, Guangzhou Xinhua University, Guangzhou, 510520 China

**Keywords:** transforming growth factor-beta receptor, Smad, transactivation dependent, GPCR signalling, Akt, phospho-Smad

## Abstract

The Smad transcription factors are well known for their role at the core of transforming growth factor-β (TGF-β) signalling. However, recent evidence shows that the Smad transcription factors play a vital role downstream of other classes of receptors including G protein-coupled receptors (GPCR). The versatility of Smad transcription factors originated from the two regions that can be differently activated by the TGF-β receptor superfamily or through the recruitment of intracellular kinases stimulated by other receptors classes such as GPCRs. The classic GPCR signalling cascade is further expanded to conditional adoption of the Smad transcription factor under the stimulation of Akt, demonstrating the unique involvement of the Smad transcription factor in GPCR signalling pathways in disease environments. In this review, we provide a summary of the signalling pathways of the Smad transcription factors as an important downstream mediator of GPCRs, presenting exciting opportunities for discovering new therapeutic targets for diseases.

## Introduction

Cellular signalling is the process through which hormones, cytokines, growth factors and environmental stimuli elicit their effects to alter or determine the phenotypic state of cells. G protein-coupled receptors (GPCRs) are the largest class of membrane receptors [1]. Thus, unsurprisingly, they regulate multiple physiological and pathophysiological processes. The therapeutic potential of targeting GPCRs or related signalling intermediates is evident with the fact that GPCRs are the largest family of targets for approved drugs, occupying approximately 30% of all FDA and EMA-registered drugs [2].

The activation of receptors can stimulate an array of signalling molecules (“second messengers”) that determine biological outcomes. As such, the receptors and their signalling cascades are major targets for therapeutic agents. Transcription factors are the critical second messengers of receptors that control cellular responses by regulating gene transcription and protein translation. The Smad transcription factors lie at the core of transforming growth factor-β (TGF-β) superfamily receptor signalling. The TGF-β receptor superfamily are serine/threonine kinase receptors that include the TGF-β receptor (TGFBR) [3], bone morphogenic protein receptor (BMPR) [4], activin receptor [5] and nodal receptor [6]. The original role of Smad transcription factors, specifically the phospho-Smads, was identified from studies of ^32^P-ATP labelling of TGF-β-treated cells by Macias-Silva [7] and Eppert and colleagues [8]. The early work in defining the TGF-β signalling pathway was undertaken by Derynck and colleagues from the University of California, San Francisco [9, 10] and Massague and colleagues from the Sloan Kettering Institute in New York [11–14]. The Smad transcription factors (Smads 1–9) are divided into three distinct sub-groups: receptor-regulated Smads (R-Smads) (Smad 1, 2, 3, 5, 8 and 9), common mediator Smad (Co-Smad) (Smad 4) and the inhibitory Smads (I-Smads) (Smad 6 and 7). The activation of TGF-β superfamily receptors leads to R-Smad phosphorylation, which forms an oligomer with a co-Smad and other co-factors before travelling to the nucleus to regulate the expression of genes and cellular responses [15]. As evolution has unfolded, intricate cellular studies have uncovered Smad signalling pathways that extend beyond TGF-β signalling [16]. These exceedingly interesting and somewhat unexpected findings show that the Smad transcription factors are pivotal in regulating biological responses downstream of GPCRs [17–21], toll-like receptors [22, 23], and tyrosine kinase receptors (TKR) [24, 25], suggesting that Smad transcription factors regulate cellular responses beyond those associated with activating TGF-β superfamily receptors.

**Fig. 1:**
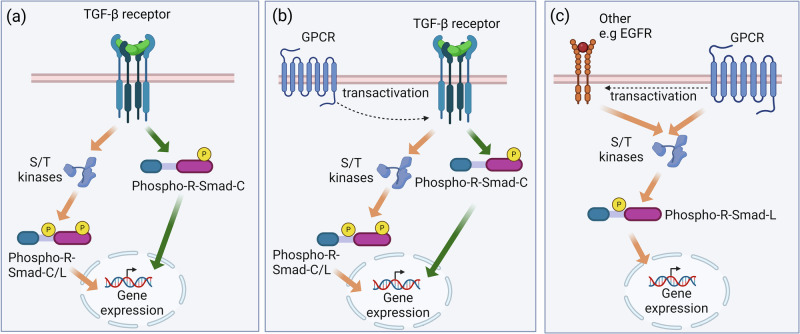
GPCR dependent and independent Smad signalling. **a** The activation of the TGF-β superfamily receptor leads to the phosphorylation of R-Smads in the carboxyl terminal (Phospho-R-Smad-C) which can travel to the nucleus to regulate gene expression. The activation of TGF-β superfamily receptors also results in the recruitment of serine/threonine kinases (S/T kinases) to phosphorylate the linker region of R-Smads (Phospho-R-Smad-L), phosphorylated R-Smad-C/L can also travel to the nucleus to regulate gene expression. **b** GPCR via transactivation of the TGF-β superfamily receptors phosphorylate R-Smad-C which travels to the nucleus to regulate cellular responses by modulating gene expressions. The activation of TGF-β superfamily receptor also result in the recruitment of S/T kinases to phosphorylate the linker region of R-Smads which have carboxyl-terminal phosphorylated. **c** R-Smads are activated by GPCRs through the recruitment of S/T kinases, which phosphorylates the linker region of R-Smads. The R-Smads with only linker region phosphorylated then translocate to the nucleus to regulate transcription and cellular responses. Figure created in biorender.com.

Smad transcription factors, per se, are implicated in many physiological and pathophysiological processes [26]. Smad transcription factors are biochemically comprised of the N-terminal, C-terminal and a central “linker region”, the final of which contains serine/threonine residues that can be phosphorylated [27, 28]. The canonical principles of Smad signalling described almost three decades ago outline that TGF-β or BMP activate their respective cognate receptors, TGFBR or BMPR, which directly phosphorylate R-Smads at the carboxyl-terminal [3, 29]. The phosphorylated R-Smads form a Smad transcription factor complex with multiple and various other transcription factors to regulate gene transcription [15]. Activation of TGF-β superfamily receptors also leads to the employment of intracellular serine/threonine kinases, which phosphorylate specific serine/threonine residues in the linker region of R-Smads, termed non-canonical Smad signalling (Fig. 1a) [26, 30–32]. Non-canonical Smad signalling is not unique to the TGF-β signalling pathway as many cytokines [24, 25, 33, 34], peptide hormone [21], and abnormalities, such as increased shear stress [35], helicobacter infection [36] and melanoma [31, 37] can also activate intracellular serine/threonine kinases, resulting in the phosphorylation of the linker region of R-Smads. As a further level of complexity, the TGFBR or BMPR can be activated via receptor-receptor transactivation-dependent mechanisms [38]. Transactivation-dependent signalling occurs where a receptor, such as a GPCR, acts via intricate cell membrane mechanisms to (trans)activate the TGF-β superfamily receptors, showing that Smad transcription factors are an important mediator of GPCR signalling pathways [17, 31, 39, 40].

## Mechanisms of R-Smads activation by GPCRs

The current paradigm of GPCR signalling covers three major pathways. Firstly, the classical pathway in which ligand engagement causes G protein binding and downstream signalling [41, 42]; secondly, the β-arrestin pathway of signalling via ligand-regulated scaffolds [43, 44]; and thirdly, as initially described by Ullrich and colleagues [45, 46], the transactivation of TKR. Receptor transactivation is defined as the mechanism by which the agonist occupancy of its cognate GPCR leads to the activation of a second cell surface receptor in a relatively short time (seconds to minutes) in the absence of de novo protein synthesis [47]. The GPCR transactivation-dependent signalling of TKRs was later broadened to include GPCR transactivation of the TGF-β superfamily receptor signalling pathway [40, 48, 49]. We [18, 47, 50, 51] and others [52–55] showed that multiple GPCR agonists can transactivate the TGF-β superfamily receptors to mediate biological effects via phosphorylation of R-Smads. A genome-wide study revealed that almost 50% of the genes regulated by the GPCR, protease-activated receptor (PAR)-1, were dependent on the transactivation of epidermal growth factor receptor (EGFR) or TGFBR, demonstrating that the downstream responses of GPCR are prominently a result of transactivation dependent signalling [56]. In GPCR transactivation-dependent signalling, the transcriptional responses initiated by a GPCR may be downstream responses of another signalling pathway. This allows for the modulation of the disease-causing pathways by targeting the secondary signalling pathways, leaving the physiological response of the GPCR unaffected.

There are several pathways through which Smads are involved in GPCR signalling. Firstly, the well-characterised GPCR transactivation of the TGF-β superfamily receptors leads to the phosphorylation of R-Smads, and the cell then behaves qualitatively identically to one receiving direct activation of the TGF-β superfamily receptors (Fig. 1b) [17, 39, 47, 57]. Certain GPCR agonists under basal conditions do not induce R-Smad carboxyl-terminal phosphorylation. However, TGFBR availability on the cell surface can be enhanced when Akt kinase is activated, allowing the active TGF-β liberated from GPCR activation to bind to the TGFBR, rendering cells responsive to GPCR transactivation signalling via R-Smad carboxyl-terminal phosphorylation [58]. Under these final conditions, carboxy-terminal phosphorylation of R-Smads occurs with consequent onward downstream signalling. Secondly, GPCRs can recruit a plethora of intracellular serine/threonine kinases that can phosphorylate Smad2 in the linker region, activating the transcriptional activity of Smad2 [22, 24, 26, 59–64]. Intriguingly, the phosphorylation of Smads in the linker region can regulate cellular responses without Smad carboxyl-terminal phosphorylation [34], showing that activation of TGFBR is not necessary for the activation of Smad transcription factors to regulate gene transcription, thus allowing us to hypothesise that the Smad linker region is a signalling pathway in its own right and not simply a pathway for regulating canonical TGFBR signalling (Fig. 1c) (reviewed in [65]).

### The activation of R-Smads by GPCR via the transactivation of TGF-β superfamily receptors

GPCR transactivation of the TGF-β superfamily receptors leads to the phosphorylation of R-Smads, which function as a transcription factor to regulate gene expression (Fig. 1b). The serine protease and GPCR agonist, thrombin, signals via its respective GPCR—PAR-1, to stimulate phospholipase C/protein kinase C and an increase in intracellular calcium but also stimulates signalling pathways that regulate Smad2 phosphorylation [17, 66, 67]. In human vascular smooth muscle cells (VSMCs), thrombin via PAR-1 transactivates the TGFBR, stimulating Smad2 carboxyl-terminal phosphorylation (pSmad2C) [17, 67, 68]. Functionally, PAR-1 transactivation of TGFBR in human VSMC leads to the modification and elongation of the glycosaminoglycan (GAG) chains on the lipid-binding proteoglycans, contributing to the development of atherosclerosis [17, 39, 67, 69].

TGF-β is produced in a latent form and requires activation before eliciting cellular actions [38, 70]. The activation of the latent TGF-β is achieved through various mechanisms, including integrins [71], metalloproteases [23] and reactive oxygen species [72]. RGD-binding integrins (αvβ1, αvβ3, αvβ5 and αvβ6) mediate the release of active TGF-β via ROCK signalling and cytoskeleton reorganisation, which creates a physical force to unwind the latent TGF-β complex to liberate active TGF-β, allowing the free, active TGF-β to bind to and activate the TGFBR [71, 73]. Thrombin stimulation of pSmad2C is blocked by an RGD peptide, indicating PAR-1 mediated TGFBR activation is dependent on ROCK/RGD-binding integrins [69]. The model of PAR-1 initiated ROCK signalling pathway and RGD integrin-mediated TGFBR activation is also observed in other cell types such as mouse epithelial cells [71], liver fibroblasts [74], colon adenocarcinoma cells [75] and fibrosarcoma cells [75]. Another agonist of the PAR-1 signalling pathway—factor Xa, also transactivates TGFBR in mouse fibroblast [76]. Smad2 is not the only R-Smad activated by PAR-1 transactivation of TGFBR. In gingival fibroblasts, thrombin via PAR-1 transactivated the TGFBR to stimulate Smad3 carboxyl-terminal phosphorylation (pSmad3C) via αVβ1 [52]. Thrombin transactivation of the TGFBR also results in the recruitment of serine/threonine kinases to stimulate the phosphorylation of Smad2 in the linker region (pSmad2L) [39, 77]. There are at least four residues that can be phosphorylated in the linker region of the Smad2—threonine residue (Thr220) and three serine residues (Ser245/250/255) [27] and in the Smad3—threonine residue (Thr179) and three serine residues (Ser204/208/213) [78]. In human proximal tubular epithelial cells, the PAR-2 agonist—2f-LICGRLO-NH_2_ stimulated the Smad2 and Smad3 linker region phosphorylation (pSmad2/3 L) [77, 79]. Mechanistic studies reveal that PAR-2-mediated pSmad2L occurs via transactivation of the TGFBR, and pSmad2L is responsible for pro-fibrotic connective tissue growth factor (CTGF) expression. Silencing of PAR-2 in a unilateral ureteral obstruction-induced kidney disease model reduced pSmad2L and CTFG mRNA expression in kidneys compared to wild-type control, demonstrating that targeting the non-canonical Smad signalling pathway represents a potential therapeutic role for treating renal fibrosis [77]. Thrombin-mediated pSmad2L demonstrates a high level of control, with specific kinases being used to regulate the phosphorylation of different residues of the Smad2 linker region. In human VSMCs, thrombin-mediated phosphorylation of the Thr220 depends on p38, Jnk and PI3K, while the phosphorylation of the serine residues includes p38, Erk, and PI3K [59]. Inhibition of Jnk does not affect thrombin-mediated phosphorylation of the serine residues [59]. The pSmad2L activated by PAR-1 in human VSMC is associated with the expression of a multitude of genes related to the modification of GAG chain structure on lipid-binding proteoglycans [80–82]. Specifically, the phosphorylation of the serine residues was associated with genes involved in the elongation of GAG chains [22], whereas the activation of the Smad2 linker threonine residues was associated with the expression of genes associated with the initiation of GAG chain synthesis on proteoglycan core proteins [22, 59], showing that Smad2 is a highly specific transcription factor capable of using the phosphorylation of different positions in the Smad2 linker region to process the signals from different mediators to control the expressions of multiple, but specific, genes.

PAR-2-mediated pSmad2L is regulated by PI3K and Erk-dependent pathways and correlated with CTGF expression [77]. PAR-2 can transactivate TGFBR. However, TGFBR also transactivates the PAR-2 signalling pathway to produce pSmad3C in human pancreatic duct epithelial cancer cells (PANC-1), human metastatic pancreatic adenocarcinoma cells (Colo357, IMIM-PC1) and HaCaT cells [83].

Lysophosphatidic acid (LPA) has at least 6 GPCRs (LPAR1-6) from which a signal can be activated [84, 85]. LPAR transactivation of the TGFBR leading to pSmad2C has been described in several cellular models [18, 86, 87] with slight variations in the signalling cascade. In mouse epithelial fibroblasts, LPA signals via LPAR2, RhoA/ROCK and integrin αvβ6-dependent pathways to activate the TGFBR and downstream pSmad2C [87]. LPA-mediated pSmad2C leads to the synthesis of fibrogenic genes via Gαq-dependent pathways [87]. Similarly, in mouse kidney proximal tubule cells, LPA signals through LPAR2 to activate Gαq to transactivate the TGFBR in a RhoA/ROCK and αvβ6 dependent manner to stimulate secretion of platelet-derived growth factor-B and CTGF [86]. Although the predominant cascade was similar, different LPARs and integrins are employed to relay the signals from LPAR to TGFBR. In human airway smooth muscle cells isolated from asthmatic and non-asthmatic patients, αvβ5 instead of αvβ6 is used in the LPA-mediated mechanism to induce the activation of the TGFBR, leading to Smad2/3 phosphorylation [54]. In human VSMCs, although LPAR1, LPAR2 and LPAR5 are abundantly expressed, only LPAR5 can transactivate the TGFBR to stimulate pSmad2C again demonstrating the specificity of these pathways [18]. LPAR5-mediated pSmad2C occurs via ROCK-dependent pathways leading to the stimulation of genes involved in GAG chain synthesis of lipid binding proteoglycans [18] and monocyte chemoattractant protein-1 [88]. Conditioned media from ascites fluid of patients with ovarian cancer was shown to stimulate pSmad2C, cell differentiation and an increase in stromal cell-derived factor-1 in human adipose tissue-derived mesenchymal stem cells. These responses were blocked by LPAR1/2/3 and TGFBR inhibitors, demonstrating that cancer-derived LPA signals via TGFBR/Smad signalling pathways [89]. In keratinocytes, LPA-mediated cell migration and cell proliferation are dependent on Smad3 phosphorylation which is inhibited by a TGFBR inhibitor [55]. The mechanism of LPAR transactivation of TGFBR is also observed in human corneal fibroblasts [53], as treatment with LPA stimulated pSmad3 but not SpSmad2 leading to TGF-β protein expression. In myoblasts, inhibiting the TGFBR or silencing Smad2/3 inhibits LPA-stimulated CTGF expressions. LPA recruits Jnk but not Erk to stimulate CTGF expression, suggesting that LPA-induced CTGF expression may potentially be occurring via TGFBR activation and is regulated by pSmad2/3 L [90].

In human VSMC, endothelin-1 (ET-1) transactivates the TGFBR with profound cellular specificity; although two ET-1 receptors, ET_A_ [91] and ET_B_ [92], are expressed in human VSMCs, only ET_A_ is used by ET-1 to relay signals to TGFBR. ET-1 via ET_A_ stimulated pSmad2C to regulate the synthesis of the GAG chain synthesis and the retention of LDL, contributing to atherosclerosis [51, 93]. The ET-1 mediated pSmad2C was inhibited by the NOX inhibitor—diphenyliodonium (DPI) and the ROS scavenger—*N*-acetyl-*L*-cysteine (NAC), which correlates with a reduction in the mRNA expression of GAG chain synthesising genes [51, 94, 95], demonstrating that in human VSMC, ET_A_ transactivates the TGFBR via ROS/NOX-dependent pathways to regulate GAG chain modification [51]. In contrast, ET_B_ was activated in bovine aortic endothelial cells to transactivate TGFBR, which occurs via the rearrangement of the cytoskeleton initiated by ROCK-dependent pathways [96, 97]. Interestingly, this ET-1-mediated pSmad2C is inhibited by cycloheximide, showing that in bovine aortic endothelial cells, ET-1-mediated TGFBR required de novo protein synthesis [97]. The differences observed between the transactivation cascade in endothelial cells and VSMCs may be attributable to the different GPCRs involved (ET_A_ vs ET_B_), a point worthy of further investigation. Human endothelial cells isolated from healthy controls or patients with systemic sclerosis showed an increase of pSmad2C when treated with ET-1, which was inhibited by a dual ET-1 receptor inhibitor, macitentan [98]. In human VSMC, ET-1 stimulated pSmad2L via NOX/NAPDH and ROS, resembling the ET_A_ transactivated TGFBR signalling pathways, correlating with the increased mRNA expression of GAG synthesising genes [21]. ET-1 mediated pSmad2L in VSMCs via a NOX/p38 MAPK axis, consistent with earlier findings using a TGF-β agonist in human VSMCs [99]. This highlights that in human VSMC, transactivation-dependent pSmad2L is reliant on p38 MAPK. The signalling cascade associated with ET_A_ transactivation of the TGFBR leading to increased pSmad2C in human VSMC differs from what has been observed with other GPCR agonists, thrombin [17] or LPA [18], where thrombin and LPA responses are dependent on Rho/ROCK-dependent pathways.

Another GPCR that has gained traction in TGFBR transactivation is the angiotensin II (Ang II) receptor. Ang II and TGF-β are intimately associated with the pathogenesis of vascular and cardiac fibrosis [100], hypertrophy [101] and heart failure [102]. Treatment with Ang II correlates with an increase of TGF-β release and Smad2/3 phosphorylation in several models of fibrosis [103–105]. In Ang II-infused hearts, an upregulation of pSmad2C and CTGF expression was attenuated when exposed to a TGFBR inhibitor, suggesting that Ang II-induced CTGF production depends on TGF-β/Smad signalling [103]. In a rat model of microvessel fibrosis, Ang II-induced CTGF protein expression and pSmad2/3 C were attenuated with the treatment of a heptapeptide hormone Ang-(1–7). Ang-(1–7) inhibited Ang II-induced phosphorylation of Erk MAPK but did not affect the expression of TGF-β [104]. These results suggest that Ang II-mediated cardiac fibrosis occurs via TGFBR-activated MAPK/Smad-dependent pathways, and Ang-(1–7) prevents fibrosis by targeting a signalling intermediate in this pathway. Using a similar Ang II cardiac fibrosis model, the role of sodium-glucose co-transporter inhibitor, dapagliflozin, was assessed. Dapagliflozin attenuated Ang II-induced pSmad2/3 C, TGF-β, collagen I and collagen III expression [105]. These results demonstrate that dapagliflozin reduced Ang II-induced cardiac fibrosis and remodelling by inhibiting the synthesis of TGF-β, which led to a reduction in pSmad2/3 C [105].

Ang II and TGF-β dependent pathways contribute to atrial fibrosis, the underlying pathophysiology of atrial fibrillation [106]. The role of long non-coding RNA plasmacytoma variant translocation 1 (PVT1) was assessed in atrial fibrillation. Atrial muscle tissue from patients with atrial fibrillation demonstrated an increase in PVT1, which correlated with an increase in collagen I and collagen III [106]. In atrial fibroblasts, Ang II-mediated Smad2 phosphorylation was facilitated by PVT1 overexpression and attenuated with PVT1 knockdown [106]. Sp1 and miR-128-3p mimetic reversed the PVT1 overexpression and mediated the facilitation of fibroblast proliferation, collagen production, and TGF-β/Smad signalling under Ang II stimulation [106]. In a cardiac fibrosis mouse model, silencing of PVT1 attenuated Ang II-induced inflammatory infiltration and atrial fibrosis, collagen production and TGF-β/Smad activation [106]. These results demonstrated a role for the long non-coding RNA, PVT1, in Ang II-mediated Smad phosphorylation as a driver of atrial fibrosis. In addition to TGFBR, BMPR can also be activated by the Ang II receptor. Infusion of Ang II for 2 weeks in mice induces the phosphorylation of Smad1 and Smad5 in the carboxyl-terminal, leading to cardiac hypertrophy, which is inhibited by BMPR2 inhibitor, LDN193189 [107]. Mice with BMPR2 knockout showed reduced Ang II-induced cardiac tissue growth, demonstrating that Ang II can transactivate BMPR2 to activate Smad1 and Smad5, resulting in cardiac hypertrophy [107].

5-Hydroxytryptamine (5-HT) (or serotonin) is a molecule that has diverse biological responses, controlling neurotransmission [108], peripheral and cerebral vascular tone [109] and gastrointestinal muscle contraction [109]. Apart from 5-HT_3_, an ion channel, 5-HT signals by binding to cognate GPCRs [108]. In pulmonary artery VSMCs, 5-HT through the 5HT_1B/1D_ receptor transactivates BMPR1A to induce Smad1/5/8 phosphorylation in the carboxyl-terminal and promotes the translocation of Smad1/5/8 into the nucleus via the ROCK-dependent signalling pathway [40]. Infusion of 5-HT into mouse lungs induces Smad1/5/8 phosphorylation but not Smad2/3 [40]. Specific 5-HT receptor inhibitors inhibit R-Smad phosphorylation. In rats treated with thioacetamide to induce liver fibrosis, sapogrylate, a 5-HT_2A_ receptor antagonist, inhibited pSmad2/3 C and TGF-β expression in the liver tissue [110]. In mice with induced liver fibrosis, mirtazapine, also a 5-HT_2A_ inhibitor, prevented the Smad3 phosphorylation and TGF-β expression in the liver tissue [111], although the antibody used to detect Smad3 phosphorylation was not specified. The signalling pathway likely resembles the observation of pSmad2/3 C in the rat model of liver fibrosis [110]. No mechanistic studies have been undertaken to investigate the signalling cascades upon 5-HT receptor activation. More experiments are warranted to explore whether or not 5-HT receptors also employ ROCK/integrins or NOX/ROS, leading to TGFBR or BMPR activation.

In a mouse open fracture model, Smad1/5/8 carboxyl-terminal phosphorylation is enhanced but not Smad2/3 upon introducing parathyroid hormone. Parathyroid hormone, a GPCR agonist, signals via cAMP and protein kinase A to induce Smad1/5/8 carboxyl-terminal phosphorylation [112]. The administration of isoproterenol at 150 mg/kg stimulates pSmad2/3 C in rat coronary blood vessels, which is associated with an increase in vascular fibrosis. At the concentrations used in this study, isoproterenol acts as an α_1_-adrenergic receptor agonist, therefore isoproterenol-stimulated pSmad2/3 C is inhibited by tamsulosin, an α_1_-adrenergic receptor antagonist [113]. In primary fibroblast, sphingosine-1-phosphate (S1P) activates S1P_1_ and S1P_3_ receptors to induce pSmad3C via Gi/o, which then forms a transcription complex with Smad4, resulting in increased cell migration [114]. The S1P-induced cell migration is inhibited by the knockout of Smad3 [114], showing that Smad3 activation is a result of TGFBR transactivation initiated by the S1P receptor. The transactivation of TGF-β superfamily receptor by GPCRs is summarised in Table 1.

**Table 1:** R-Smads activated by GPCR agonists via TGF-β superfamily receptor transactivation.

GPCR Agonist	Phosphorylated site on R-Smads	Cell type	Mechanisms	Refs.
ET-1	Smad2 carboxyl-terminal	Human VSMCs	ET_A_ transactivation of the TGFBR via c-Abl kinase and ROS/NOX-dependant pathways	[51, 93–95]
	Smad2 linker	Human VSMCs	ET-1 via transactivation of the TGFBR downstream activation of NOX and p38-dependent pathways	[21]
	Smad2 carboxyl-terminal	Bovine aortic endothelial cells	ET_B_ transactivation of the TGFBR via ROCK and cytoskeleton pathways which requires de novo protein synthesis	[97]
Thrombin	Smad2 carboxyl-terminal	Human VSMCs	PAR-1 transactivation of TGFBR via ROCK- and RGD-integrin-dependent pathways	[17, 39, 67–69]
	Smad2	Mouse epithelial cells	PAR-1 via RhoA and Rho kinase to induce αvβ6 mediated TGFBR activation	[71]
	Smad2 linker	Human VSMCs	‐ PAR-1 transactivation of TGFBR ‐ Phosphorylation of the Thr220 occurs via p38, Jnk and PI3K ‐ Phosphorylation of the serine residues occurs via p38, Erk and PI3K	[17, 39, 59]
	Smad3 carboxyl-terminal	Gingival fibroblasts	PAR-1 transactivation of the TGFBR via αVβ1-dependent pathways	[52]
Factor Xa	Smad2	Mouse fibroblasts	PAR-1 transactivation of TGFBR	[76]
2f-LICGRLO-NH_2_	Smad2/3 linker	Human proximal tubular epithelial	PAR-2 transactivation of the TGFBR and regulated by PI3K- and Erk-dependent pathway	[77]
LPA	Smad2 carboxyl-terminal	Mouse epithelial fibroblasts	LPAR2 recruits Gαq to stimulate RhoA/ROCK and ανβ6 to transactivate TGFBR	[87]
	Smad3 carboxyl-terminal	Mouse kidney proximal tubule cells	LPAR2 recruits Gαq to stimulate RhoA/ROCK and ανβ6 to transactivate TGFBR	[86]
	Smad2 carboxyl-terminal	Human mesenchymal stem cells	LPAR1/2/3 transactivation of the TGFBR	[89]
	Smad2/3	Airway smooth muscle cells	LPA transactivation of the TGFBR through the ανβ5-dependent pathway	[54]
	Smad2 carboxyl-terminal	Human VSMCs	LPAR5 via ROCK-dependent transactivation of the TGFBR	[18, 88]
	Smad3	Human keratinocytes	LPA via transactivation of the TGFBR	[55]
	Smad2/3	Human corneal fibroblasts	LPA via LPAR1/2/3 transactivates the TGFBR	[53]
Ang II	Smad2	Human atrial fibroblasts	PVT1 facilitates the Ang-II-induced TGF-β1/Smad signalling activation via miR-128-3p/Sp1 axis.	[106]
5-HT (serotonin)	Smad1/5/8 carboxyl-terminal	Human pulmonary artery VSMCs	5HT_1B/1D_ receptor via ROCK-dependent pathways transactivates BMPR1A	[40]
Parathyroid hormone	Smad1/5/8 carboxyl-terminal	Mice bone marrow mesenchymal stem cells	PTH via cAMP/PKA and CREB pathways transactivates the BMPR2	[112]
S1P	Smad3 carboxyl-terminal	Human primary fibroblasts	S1P_1_ and S1P_3_ via G_i/o_ dependent pathway to induce TGF-β superfamily receptor activation	[114]

**Fig. 2:**
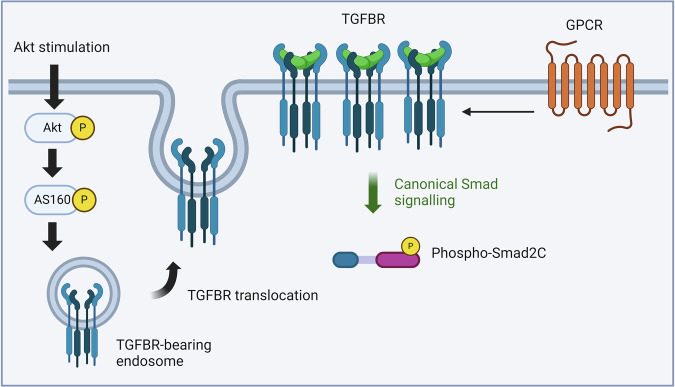
Akt acts as a switch for translocation of cytosol TGF-β receptors (TGFBR) to the membrane and GPCR transactivation dependent signalling. Activation of Akt pathways leads to the phosphorylation of AS160, which promotes the translocation of intracellular TGFBR receptors to the cell membrane. When GPCRs are activated, the transactivation of TGFBR is observed, leading to the phosphorylation of Smad2 in the carboxyl-terminal (Phospho-Smad2C). Figure created in biorender.com.

The GPCR-transactivation of TGFBR signalling in some cellular models is controlled by the abundance of TGFBR on the cell surface which is available for agonist binding [58]. Akt activation promotes the translocation of receptors residing in the cytoplasm to the cell surface through recruiting AS160, a Rab GTPase [115, 116]. Akt-mediated TGFBR translocation, first described by Derynck et al. [117], showed that glucose [117], insulin [116, 118], and insulin-like growth factor [118] stimulate phospho-Akt, which correlates with an increase of TGFBR abundance on the cell surface and the generation pSmad2/3 C. In human VSMCs [58], Ang II treatment alone does not stimulate an increase in pSmad2C above the basal level. However, when the cells were primed by pretreatment with the Akt activator, SC79, treatment with Ang II stimulated an increase in pSmad2C. In Akt-sensitised cells, GPCR-stimulated TGFBR activation is also observed in keratinocytes [58], where under basal conditions, thrombin does not induce pSmad2C [34]. However, pSmad2C was observed in Akt-sensitised cells [58]. Stimulation of Akt results in the increased availability of TGFBRs on the cell surface [58, 116]. As a result, GPCRs can induce more TGFBRs to elicit their transactivation responses, leading to an observable pSmad2C stimulation (Fig. 2). Akt is stimulated in several disease models: Alzheimer’s disease [119], cancers [120, 121], cardiac hypertrophy [122], and idiopathic pulmonary fibrosis [123]. When developing an in vitro model to study the involvement of R-Smads in other GPCR signalling pathways, pre-treatment of cells with Akt activator can be essential as the recruitment of R-Smads could be subject to Akt stimulation, which also reflects the complete picture of the disease model.

### The activation of R-Smads by GPCR independent of TGFBR

The phosphorylation of R-Smads in the carboxyl-terminal can only be achieved by direct phosphorylation by the kinase activity of the TGF-β superfamily receptor(s) (Fig. 1a, b). However, the recruitment of serine/threonine kinases is not specific to the TGF-β superfamily receptors. GPCR can also transactivate EGFR to phosphorylate the linker region of R-Smads by employing serine/threonine kinases [34, 77]. In human VSMCs, thrombin activates the PAR-1 signalling pathway to transactivate EGFR to stimulate the pSmad2L [39]. PAR-1 transactivation of the EGFR results in the upregulation of GAG chain synthesis and GAG synthesising gene expression [17, 39, 69]. The mechanism of PAR-1 transactivation of the EGFR occurs via a “triple membrane by-pass” process, which involves the activation of metalloproteinases to hydrolyse membrane-bound, heparin-binding EGF to activate EGFR [45, 47, 124]. PAR-1 transactivation of the EGFR occurs rapidly (within 30 min). However, PAR-1 transactivation of the TGFBR is transiently delayed to 2 h [39]. The distinct biochemical mechanisms of transactivation may explain the temporal differences in the response profiles. In breast cancer cell line, MDA-MB-231, EGF-induced Smad3 linker region phosphorylation at Ser208 via Akt without activating the TGFBR [125]. The phosphorylation of Ser208 is required for TGF-β-induced Smad3 activation and correlates with TGF-β-induced genes associated with epithelial-mesenchymal transition [125]. Another GPCR receptor that also leads to the recruitment of EGFR is the ET-1 receptor. The activation of ET-1 receptor transactivates TGFBR and EGFR, resulting in pSmad2L. The activation of the ET-1 receptor-stimulated pSmad2L was associated with an increase in GAG synthesising gene expression in human VSMCs [21]. A similar observation is seen in bovine aortic endothelial cells, where ET-1 stimulated pSmad2L is dependent on EGFR, which correlates with the increased expression of plasminogen activator inhibitor-1 (PAI-1) [126]. In VSMCs, activation of ET-1 and PAR-1 also results in the activation of TGFBR and R-Smads linker region phosphorylation, demonstrating that Smad2 can process signals from different receptors synergistically to fine-tune the target gene expression.

The activation of GPCR can induce pSmad2/3 L without activation of TGFBR. In keratinocytes, thrombin transactivates EGFR but not TGFBR, resulting in pSmad2L via Erk MAPK but not p38 MAPK or Jnk MAPK [34]. Thrombin-stimulated cell proliferation and PAI-1 expression correlate with EGFR activation and the recruitment of Erk MAPK, suggesting that thrombin-stimulated cellular response is controlled by pSmad2L [34]. The PAR-2 signalling pathway also results in pSmad2L in keratinocytes via EGFR and Erk MAPK, resulting in PAI-1 mRNA expressions [34]. In human tubuloepithelial cells, Ang II stimulated Smad2 phosphorylation, which was inhibited in the presence of MAPK antagonists to Erk, p38 and Jnk [127]. The Ang II stimulated expression of vimentin is independent of TGFBR and is inhibited when Smad7 is overexpressed, indicating that Ang II induced epithelial-mesenchymal transition marker expression is a result of pSmad2L via the non-canonical Smad signalling pathway [127]. In rats infused with Ang II, enhanced Smad2 phosphorylation is observed in the aorta. Pharmacological inhibition of Ang II receptors shows that Ang II-induced Smad2 phosphorylation is initiated by AT_1_ but not AT_2,_ similar to the specificity observed with ET receptors. The Ang II-induced Smad2 phosphorylation is independent of TGFBR and is mediated by p38 MAPK, supporting the downstream signalling pathway of Ang II-induced pSmad2L [20]. Ang II-induced expressions of CTGF and fibronectin are dependent on Smad7 [20]. A similar observation is seen in rat kidney tubular epithelial cells, whereby the Ang II-induced Smad3 phosphorylation by activating AT_1_ to recruit p38 and Erk in the absence of TGF-β stimulation, which correlates with CTGF and collagen 1 mRNA expression. The expression of CTGF and collagen 1 mRNA were dependent on Smad7, showing that pSmad2L is recruited by Ang II to mediate cellular responses in the absence of TGFBR activation [128]. GPCR initiated R-Smads linker region phosphorylation, which requires the recruitment of serine/threonine kinases. Currently, evidence of the GPCR recruitment of serine/threonine kinases is concentrated in the GPCR transactivation of EGFR. However, the involvement of other serine/threonine kinase receptors should be explored. Platelet-derived growth factor receptor [24] and hepatocyte growth factor receptor [129] have been demonstrated to induce R-Smad linker region phosphorylation without the activation of TGFBR. Therefore, further experiments investigating the participation of serine/threonine receptor in GPCR induced R-Smad linker region and the related downstream responses are warranted.

## Concluding remarks

GPCRs are the largest class of targets for therapeutic agents covering all aspects of diseases from cancer to fibrosis to cardiovascular disease and asthma [130–133]. The original paradigm of GPCR signalling covered classic linear signalling [42], and this was expanded three decades ago to include GPCR transactivation of TKR [46]; more recently this area was expanded further to include the transactivation of TGFBRs [67, 95, 134]. TGF-β signalling is also a massive area of biology and medicine related to many diseases, especially cancers and fibrosis [13, 135]. The discovery of GPCR transactivation of the TGF-β superfamily receptors brought Smad transcription factors into the overall paradigm of GPCR signalling. Surprisingly, intense studies of GPCR transactivation signalling revealed that GPCRs could also invoke the involvement of Smad transcription factors without the involvement and engagement of TGFBR [62]. This TGFBR independent- signalling involving Smad transcription factors occurred through the activation of intracellular serine/threonine kinases and phosphorylation of the linker region of R-Smads, nuclear translocation of the linker region phosphorylated Smad complex and modulation of gene transcription. R-Smad carboxyl-terminal phosphorylation has been reported as the pre-requisite for the formation of Smad transcription factor complexes to modulate gene expression. Therefore, molecular studies of dominant negative serine to alanine mutant transcripts to determine if either or both linker region and carboxy-terminal phosphorylation are necessary for GPCR-activated R-Smads, specifically addressing the question of what controls cellular and nuclear localisation of R-Smads. GPCRs employ diverse mechanisms to recruit Smad transcription factors to mediate downstream responses. Studying the involvement of the Smad transcription factor in the GPCR signalling pathway can provide new opportunities understanding of disease processes and discoveries of new therapeutic targets.
